# Quantification and comparison of the regional acceleratory phenomenon in bone following piezosurgery or bur osteotomy: A pilot study in rats

**DOI:** 10.1002/cre2.689

**Published:** 2022-11-11

**Authors:** Massimo Di Battista, Jeremy Kernitsky, Elias Exarchos, Taisuke Ohira, Serge Dibart

**Affiliations:** ^1^ Department of Periodontology and Oral Biology, Henry M. Goldman School of Dental Medicine Boston University Boston Massachusetts USA

**Keywords:** Corticotomy, deep learning, Orthodontics, Piezocision

## Abstract

**Background/Objective:**

The Regional Acceleratory Phenomenon (RAP) can be induced surgically via decortication (selective cortical penetrations) of bone to accelerate orthodontic tooth movement. Few studies have compared the impact and efficiency of different decortication methods to induce the RAP. The aim of this study was to determine if there is a significant difference in the intensity of the RAP induced by a surgical defect created either using a piezoelectric knife or a rotary bur.

**Methods:**

Twenty‐two Sprague–Dawley rats were divided into two treatment groups (each *n* = 8) and a control group (*n* = 6). The treatment groups were subjected to transcortical penetrations (TP) of the right tibia using either a piezoelectric knife (PTP) or a rotary bur (BTP). The right tibias of the control group animals had reflection of tissues (SHAM) and the left legs were kept for comparison (INTACT). The animals were killed at 7 and 14 days after the operation in an equally distributed manner. Microcomputed tomography images were obtained and analyzed utilizing artificial intelligence for bone cortical porosity (Ct.Po) locally and regionally.

**Results/Conclusion:**

Regionally, TP using a PTP induced significantly (*p* < .05, Kruskal–Wallis test) more Ct.Po than BTP or INTACT for both the 7‐ and 14‐day time points. PTP was not found to induce significantly more Ct.Po than SHAM at any time point. However, PTP induced significantly more Ct.Po than the INTACT group for each time point, while SHAM did not. The local analysis did not reveal any relevant significant differences between groups.

## 1 INTRODUCTION

The Regional Acceleratory Phenomenon (RAP) was described in 1983 by H.M. Frost as a sequence of events characterized by increased bone turnover that occurs in response to a noxious stimulus (Frost, 1983). Previous studies on the rat model demonstrated that reflection of a full‐thickness flap alone induced a transitory period of osseous demineralization (Yaffe et al., 1994). The RAP is the phenomenon underlying orthodontic tooth movement (Melsen, 1999, 2001), and stimulating it surgically may translate clinically to faster tooth movement. Many authors have described techniques where the bone is surgically injured to induce the RAP, reducing orthodontic treatment time (Dibart et al., 2009; Kim et al., 2009; Wilcko et al., 2001). The first technique based on the RAP included reflection of full‐arch, full‐thickness flaps, and extensive cortical penetrations using a rotary bur (Melsen, 2001). The main concern for both patients and clinicians with this technique was its invasive nature (Zawawi, 2015). This led to the development of minimally invasive techniques such as Piezocision, a flapless technique that consists of transcortical penetrations (TPs) using a piezoelectric knife through incisions in the mucosa (Dibart et al., 2009).

Since the clinical literature reports commensurate orthodontic movement acceleration on comparing conventional full‐thickness flap reflection and bur osteotomy with the less traumatic Piezocision (Abbas et al., 2016; Viwattanatipa & Charnchairerk, 2018), it may be conjectured that an injury from a piezoelectric knife will induce a stronger RAP than a bur injury. As the RAP is an ensemble of phenomena, there is no direct measure to quantify it. The ideal metric would be a direct indicator of remodeling of the bone that is quantifiable noninvasively in three dimensions. Cortical porosity (Ct.Po) is a potential metric to quantify the RAP because it is an indicator of bone remodeling (Cooper et al., 2016; Lloyd et al., 2008; Shigdel et al., 2015) that is quantifiable radiographically and corresponds to the transitory period of demineralization following an injury as described by Frost (1983). Surgical procedures that lead to osseous and periosteal injury, such as placement of implants or hip reaming for arthroplasty (Syed et al., 2013), have been shown to induce cortical porosity (Li et al., 2015) or in animal models, which is consistent with the formation of a bony callus and angiogenesis (Lienau et al., 2009). Cortical porosity has been used in the orthopedic literature as a marker for bone mineral density (BMD) when it is not relevant to examine the marrow compartment of the bone (Syed et al., 2013). In the present study, it was decided not to examine the marrow compartment as it does not contain any trabecular bone. Trabecular bone is only present in the epiphyseal regions of the tibia and the procedures were carried in the diaphyseal region. Surgery in the epiphyseal regions could lead to articular damage, which in turn can change the animal's behavior and affect other parameters, such as the load on the bone.

To date, the difference in RAP between these surgical techniques (bur and piezoelectric knife) has never been compared using quantitative measures. Most studies on the subject compare the effects of bur decortication or piezoelectric decortication against intact bone only, therefore not controlling for the resorptive stimulus of flap reflection. This study aimed to quantify and compare the RAP (via Ct.Po) occurring following different surgical injuries (Piezoelectric knife or bur) versus reflection of soft tissue alone as well as intact bone utilizing a rat model. We hypothesized that piezoelectric injury will induce more Ct.Po than rotary instruments and reflection of tissues alone.

## 2 MATERIAL AND METHODS

### 2.1 Surgical procedure

The study was approved by the Boston University Medical Center Institutional Animal Care and Use Committee (AN‐15682). The animals (*N* = 22) were 9‐week‐old male Sprague–Dawley rats weighing 300 g. Animals were purchased from Charles River Laboratories International, acclimated for at least 72 h before surgical procedures, and fed ad libitum. At the time of surgery, animals received an intraperitoneal injection of Ketamine (80 mg/kg) and Xylazine (10 mg/kg). Sustained‐release buprenorphine 0.3 mg/kg was administered subcutaneously for pain control.

**Figure 1:**
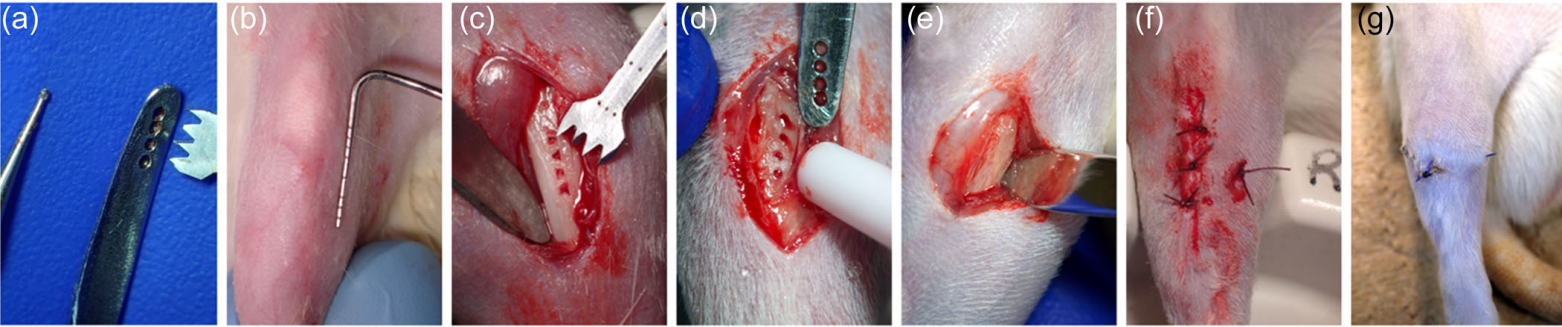
Surgical Intervention. (a) From left to right, the rotary bur, the surgical template, and the BS1 insert for the piezoelectric knife. (b) Surgical site beforeincision. The knee is used as a landmark. (c) Defect created using the piezoelectric knife. Bleeding confirms extension in the marrow area. (d) Defect created using the bur. (e) Sham procedure. (f) Closure. The total operation time was around 10 min per leg. (G) Site after 14 days of healing.

The animals were divided into three groups: PTP (Piezoelectric Transcortical Penetration), BTP (Bur Transcortical Penetration), and Control. For the PTP and BTP groups, surgical defects were created in the right tibia. For the PTP group (*n* = 8), the BS1 insert and the D1 (30 Hz modulation frequency) setting of the piezoelectric knife were used (Piezotome 2, Satelec; Acteon Group, Merignac, France). For the BTP group (*n* = 8), a carbide osteotomy bur (#½) was used at 800 rpm. A surgical template (Figure 1) ensured that the defects of the BTP group were of similar number, size, and shape as the PTP defects. The surgical defects were approximately 5 mm in length. Reflection of soft tissue was about 10 mm, to allow access to the surgical site. For both groups, the defect was extended in the marrow space and therefore completely penetrated the cortex of the bone. Cortical penetration was confirmed by bleeding from the bottom of the osteotomies. Copious and similar amounts of irrigation were used for both groups.

The control group (*n* = 6) was subjected to incision and reflection of tissues without decortication on the right tibia (SHAM). The left tibia of all animals was untouched (INTACT). Finally, the animals were killed at 7 and 14 days postoperatively in an equally distributed manner (3 at each timepoint), and µ‐CT scans were obtained for both time points.

### 2.2 Imaging analysis

µ‐CT of the affected regions was acquired using a 3D X‐ray microscope (Xradia Versa 520, Zeiss, Germany), which provided a voxel size of 6.6 µm. A beam of 80 kVp was used. The scanned regions were cylinders approximately 7 mm in height and 4 mm in diameter.

Images were segmented using a method described in the orthopedic literature (Badran et al., 2020; Reznikov et al., 2020) with a U‐net convolutional neural network (CNN), a deep learning algorithm specialized in image recognition. For ground truths, 10 slices were hand segmented in software (Dragonfly; Object Research Systems, Montreal, Québec, Canada). The data from the image slices of the µ‐CT were divided into multiple parts called patches, with 80% of the patches being used to train the algorithm, and the remaining 20% of the patches being used for validation, providing an unbiased evaluation of the accuracy of the segmentation given by the model on unseen data.

On the µ‐CT image, Ct.Po was computed as the proportion occupied by holes in the total cortical bone volume (cortical bone and holes combined). This measure of Ct.Po will be referred to as “regional cortical porosity.”

**Figure 2:**
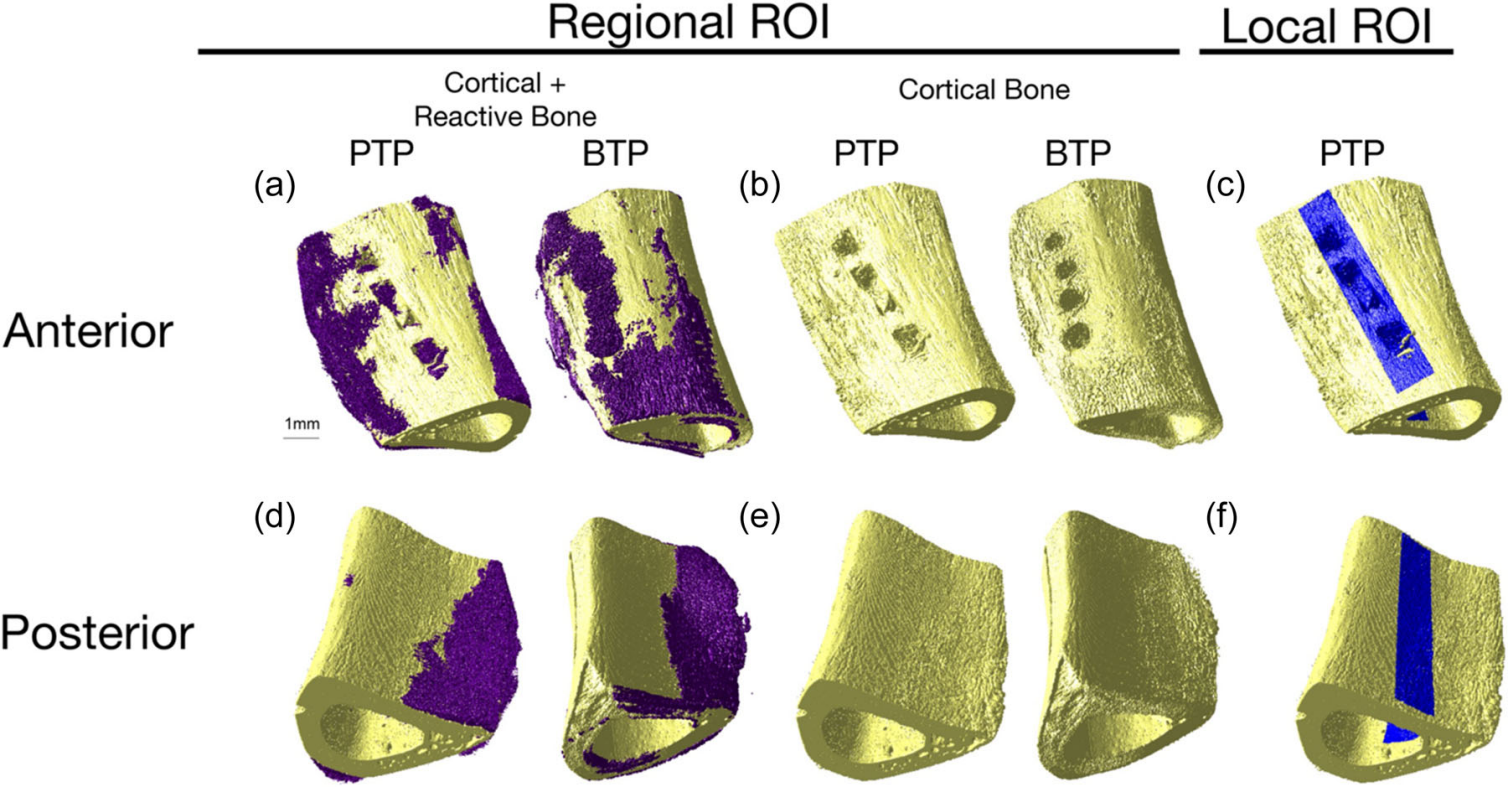
3D reconstructions of Day 7 samples. (a, d) The bone (yellow) and reactive woven calcified tissue (purple). (b, e): the bone only (yellow). (c, f): ROI for the local porosity analysis highlighted in blue (a–c) presents the anterior aspect of the bone and figures (d–f) show the posterior aspect. 3D, three‐dimensional; BTP, Bur Transcortical Penetration; PTP, Piezoelectric Transcortical Penetration.

Smaller regions of interests (ROIs) of 6 ×1 × 1 mm centered around the defects were created to observe the phenomena occurring immediately around the injury. ROIs of the same size were isolated on the opposite side of the bone to evaluate the range of demineralization. This analysis is referred to as “local cortical porosity.” The ROIs on the defect side and the opposite side are referred to, respectively, as “anterior” and “posterior.” Subscript “a” or “p” is added after a group's name to indicate that a local ROI is discuss, for example, “PTPa” for “PTP anterior.” Figure 2 shows a visual depiction of the ROIs for local and regional cortical porosity.

### 2.3 Statistical analysis

Data analysis was performed on SPSS (IBM, Armonk, NY). Data are presented as box plots, with median and quartiles. For regional and local cortical porosity, the Kruskal–Wallis test was used to assess for differences between means, and individual Kruskal–Wallis tests were used to compare each pair.

## 3 RESULTS

### 3.1 Clinical parameters

The behaviors of the rats were monitored every day for the first week, and then every 48 h. No differences in behavior and weight were observed between groups. One rat suffered from an infection of the surgical wound. The imaging analysis was inconsistent with other animals and presented large amounts of reactive tissue; therefore, the animal (PTP group, 14 days) was excluded.

### 3.2 Regional porosity

**Figure 3:**
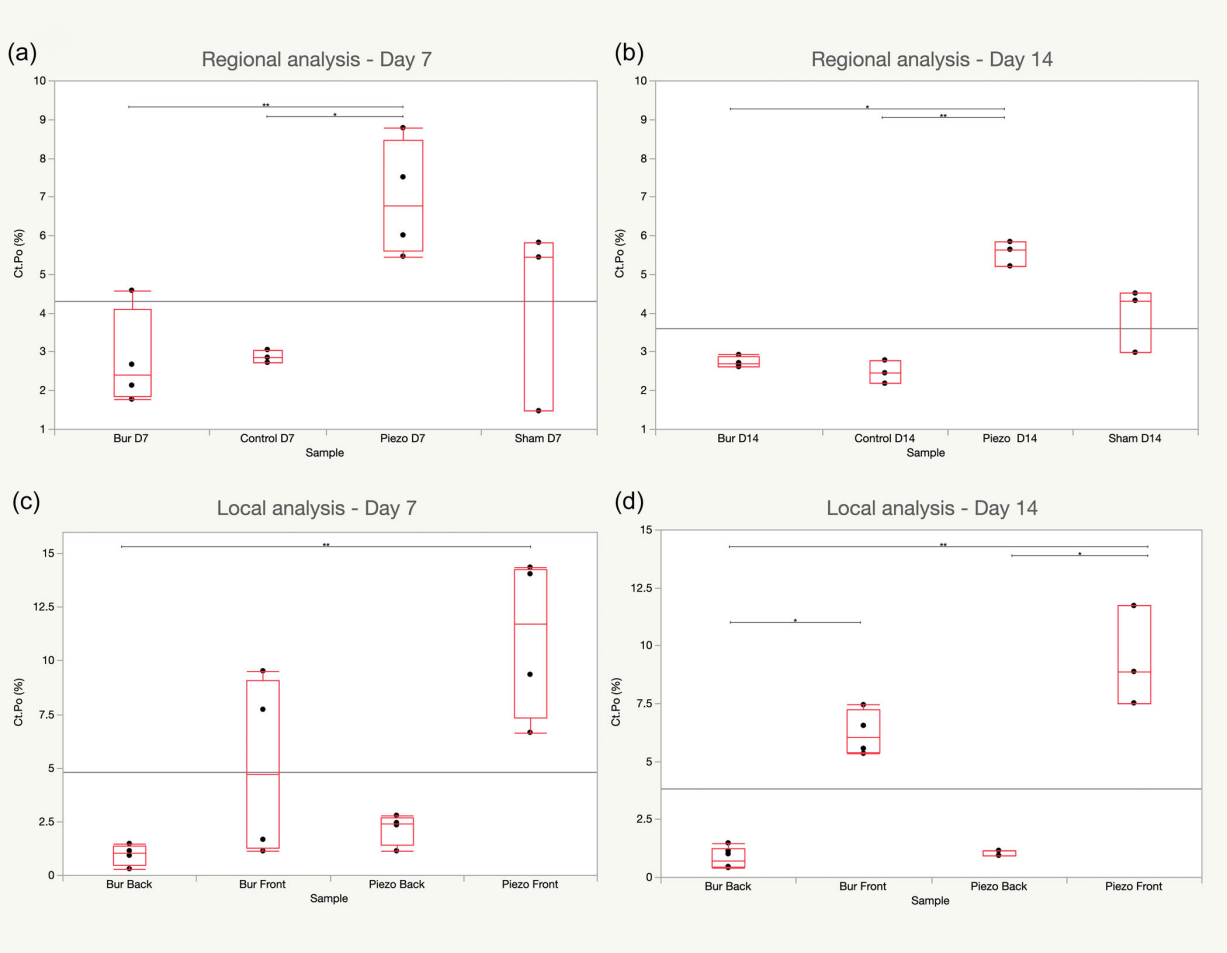
Cortical porosity of the bone. Results are presented in box plots, with means and quartiles. Statistical significance between means was assessed using an omnibus Kruskal–Wallis test and individual pairwise Kruskal–Wallis tests. *p < .05, **p < .01.

As shown in Figure 3a,b, ptp induced the most regional cortical porosity for both time points, with means of 6.94% (SD ± 1.39) and 5.56% (SD ± 0.27), respectively. It was significantly more than BTP and INTACT at Days 7 and 14.

### 3.3 Local porosity

The effects of the surgical defects on Ct.Po were mostly confined to the anterior ROIs. For Day 7, PTPa showed a mean Ct.Po of 11.10% (SD ± 3.24), which was significantly higher than BTPb (Figure 3c).

At Day 14 (Figure 3d), PTPa showed significantly more Ct.Po (mean 9.37% SD ± 1.75) than PTPb and BTPb.

BTPa showed significantly more mean Ct.Po (6.30% SD ± 0.82) BTPb.

### 3.4 Automated segmentation

Our segmentation using deep learning was successful. All models had accuracy rates of over 99% on verifying on 20% of unseen data that were retained for validation. Figure 5 shows the segmented slices.

## 4 DISCUSSION

**Figure 4:**
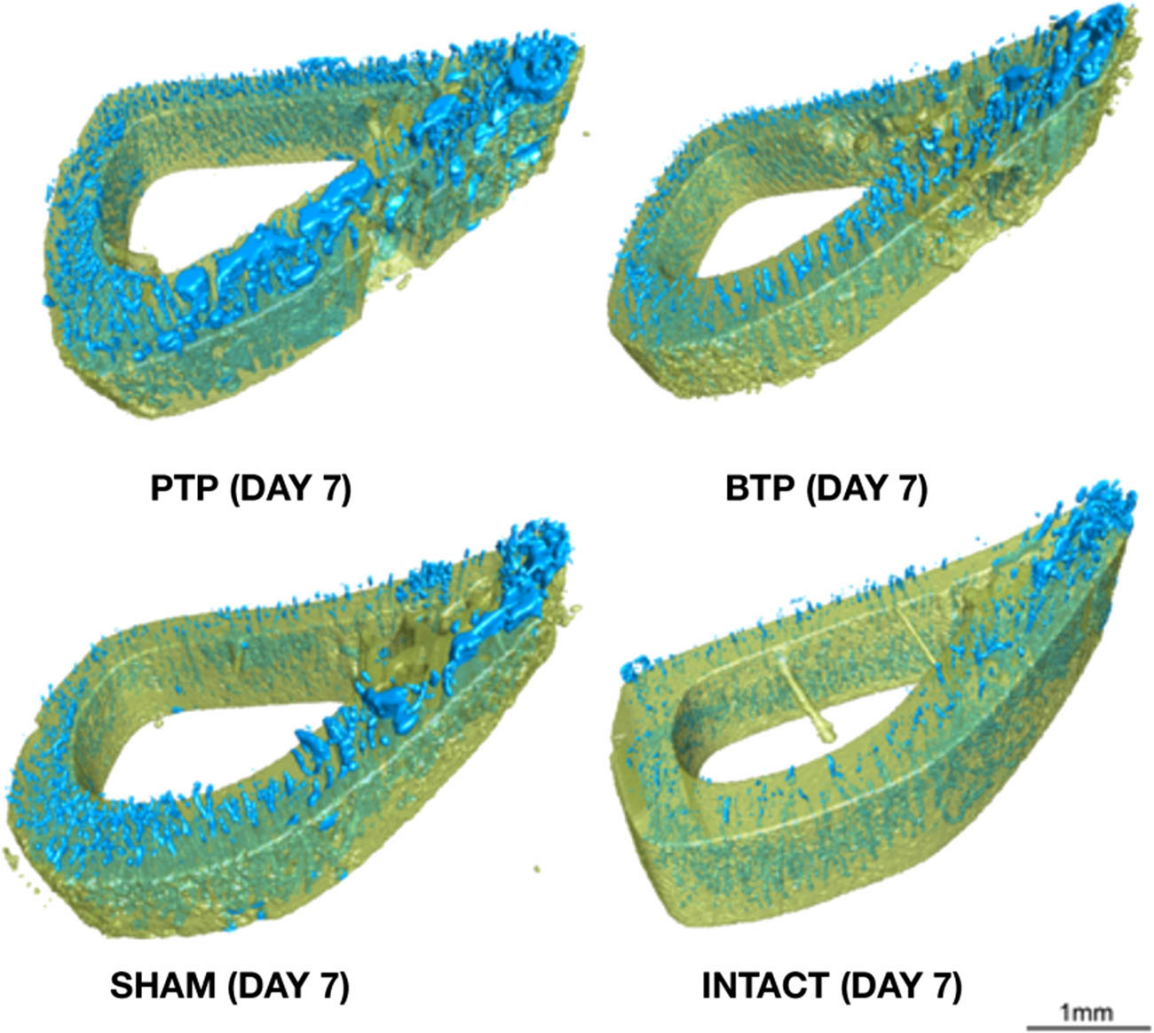
Network of porosities. 3D renderings of the bone (yellow) and porosities (blue) from the 7‐day sample. Note the vascular net‐like aspect of the structures and the marked increase in PTP. 3D, three dimensional; BTP, Bur Transcortical Penetration; PTP, Piezoelectric Transcortical Penetration.

This study compares the intensity of the RAP occurring after different corticotomy injuries on the rat model. A few previous studies had attempted to compare and quantify some parameters such as BMD and bone fill (%) between such lesions, but the µ‐CT scan resolution (20.5 μm/voxel) that they used was not precise enough to detect the porosities that we observed (Reside et al., 2015). The resolution of our images (6.6 μm) allowed for the detection of porosities as small as osteocyte lacunae (Bach‐Gansmo et al., 2015). The pore network was rendered in 3 dimensions (Figure 4) to observe the architecture of porosities. Ct.Po seems to be organized in tubular structures with what appears to be a vascular net appearance. Cooper et al. (2016) discussed Ct.Po and its relation to remodeling; bone that actively remodels presents an augmentation in porosity organized in tunnel‐like resorption spaces, similar to what we observed in the experimental groups of the present study.

**Figure 5:**
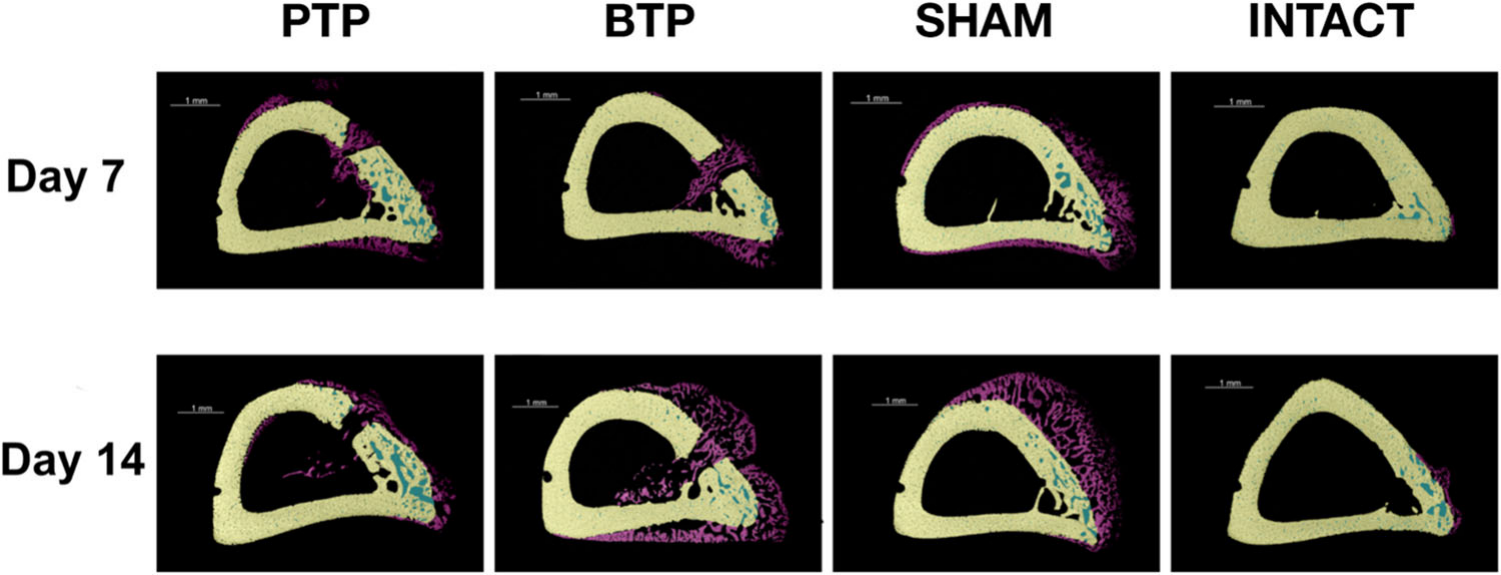
Segmentation results at different time points. Cortical bone (yellow); callus (purple), and cortical porosity (blue). Pixel size = 6.6 μm. BTP, Bur Transcortical Penetration; PTP, Piezoelectric Transcortical Penetration.

**Figure 6:**
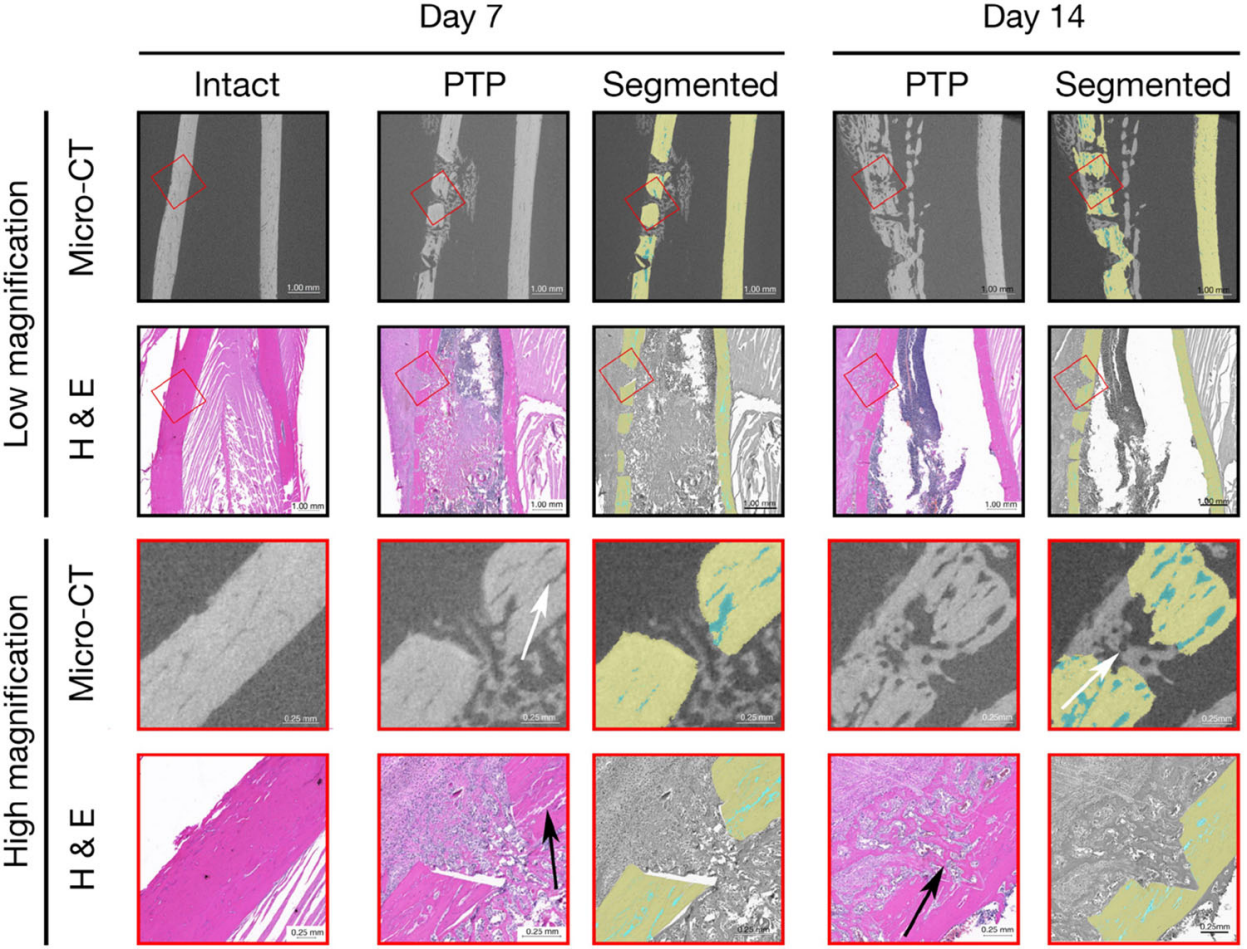
Comparison with histology from Kernitsky et al. (2021), Lienau et al. (2009). The Days 7 and 14 histological slides were segmented to compare cortical porosity with data from this study. The result was 7.21% at Day 7 and 5.41% at Day 14, which is consistent with the results from this study. Low‐magnification μ‐CT and photomicrographs are shown on the top two rows along with observed general morphology. High‐magnification areas in red boxes overlaying the low‐magnification images are displayed on the bottom rows. Arrows showing resorption areas on the 7‐day sample and bone fill on the 14‐day sample. PTP, Piezoelectric Transcortical Penetration; μ‐CT, micro‐computed tomography.

More recently, Kernitsky et al. (2021) reported large areas of resorption in bone after PTP based on histological analysis, which is consistent with our imaging analysis as well. Photomicrographs from their experiments as well as permission to use them were obtained from the authors. Their histological results are compared to the segmented slices of the scan in Figure 6. They also found that the defect had to completely penetrate the cortical layer of the bone and extend into the marrow space to induce a strong response from the bone, which is why we investigated transcortical defects in the present study. Figure 6 shows a comparison of the porosity observed on histological samples versus what is observed radiographically. Histological cortical porosity was 7.21% at Day 7 and 5.41% at Day 14, which is consistent with the imaging results from this study (6.94% at Day 7 5.56% at Day 14). For this comparison, the porosity was segmented manually on the histology slices. This was possible in this scenario because of the small amount of data to be segmented and the high contrast between the bone and the porosities observed on the photomicrographs.

Cortical porosity is challenging to quantify on µ‐CT images when a surgical defect is actively healing. As the callus undergoes calcification, it will become as radio‐opaque as the original bone and most segmentation methods will often mistake it for a porous region of cortical bone, returning a higher porosity value and therefore false‐positive results. Deep learning, a type of artificial intelligence was used because it can distinguish nuances from shape and edge information rather than only intensity difference (Badran et al., 2020) and therefore could exclude the callus based on its woven pattern instead of radio‐opacity. Reznikov et al. (2020) wrote a paper discussing deep learning segmentation techniques in which these issues are addressed. Our case resembles the “case 2” they describe, which is a case where the authors segmented bone from background noise and other materials. This technique was applicable to our project. Some of the authors of the aforementioned paper also used this technique with success for analysis of samples in material sciences and published a paper with extensive details on the methods (Badran et al., 2020).

The decision to sacrifice animals at 7 and 14 days was made to observe the early phase of healing as remodeling activity has been shown to peak during this period on the rat model (Kernitsky et al., 2021; Ohira et al., 2019; Zou et al., 2019).

The regional cortical porosity analysis includes the whole scanned volume. This analysis gives a good overview of the biological modifications in bone caused by our surgical interventions, which could help translate experimental results for clinical application, such as accelerating orthodontic movement with a surgical intervention conducive to alveolar bone demineralization. In our regional analysis, the PTP group induced significantly more Ct.Po than the BTP group at both 7 and 14 days time points, pointing toward more potent bone remodeling stimulus with piezosurgery.

Our findings suggests that the piezoelectric knife induces a stronger RAP than the bur, which implies that superior cellular activation as the RAP is a cell‐mediated phenomenon (Frost, 1983). Recent literature suggests that the ultrasonic vibration produced by piezosurgery stimulates early osteocyte apoptosis, which results in bone remodeling (Zou et al., 2019). Other studies found more interleukin‐1β, an actor in osteoclast differentiation in piezoelectric surgery sites than in bur surgery sites (Chenu et al., 1994; Lee et al., 2006; Preti et al., 2007). Piezosurgery also appears to be less damaging to the vitality of osteoprogenitor cells such as osteoblasts (Pereira et al., 2018) and osteocytes (Anesi et al., 2018) when compared with rotary instruments. In addition, it has been shown that blood supply around a surgical bone defect was better preserved with piezosurgery (Reside et al., 2015). This is consistent with our findings; at Day 7, PTP_a_ was already showing a lot more Ct.Po than its posterior counterpart, which was not the case for BTP_a_, hinting delayed bone remodeling. These results should be interpreted with caution because although the observation can be made while looking at the data, it was not statistically significant. Furthermore, animal studies have shown that BTP alone would not induce bone remodeling in the early phases of healing (Baloul et al., 2011). Regionally, PTP showed the most Ct.Po Ct. and was the only one to show significantly more porosity than INTACT for both 7‐ and 14‐day time points.

In the present study, SHAM showed higher Ct.Po than both BTP and INTACT at Days 7 and 14. These findings seem paradoxical because the RAP is said to be proportional to the magnitude and the nature of the stimulus (Verna, 2016), therefore, one would assume that the RAP would be stronger with both stimuli (reflection of tissues + bur injury) instead of only one. Although not statistically significant, these results are worth exploring in detail because the lack of significance might be owed to the small sample size and the higher dispersion of the SHAM data. The explanation may reside in noxious bone injury resulting from the use of a bur. Healing might be delayed from heat and pressure damage (Aghvami et al., 2018). Vercellotti et al. (2005) evaluated the level of the alveolar bone crest after ostectomy with piezosurgery and burs in alveolar ridges of dogs and reported bone loss at 14 days in the diamond and carbide bur groups versus bone level gain in the group treated with piezosurgery. They attributed the damage to excessive pressure on the bone during bur instrumentation. Rullo et al. (2013) conducted a randomized controlled trial comparing a conventional bur to a piezoelectric knife for the extraction of impacted third molars. The bone biopsies they obtained during surgery showed thermal bone necrosis on the bur samples only. Ma et al. (2013) conducted a histomorphometrical study comparing the bone healing after osteotomies performed by piezosurgery versus osteotomies performed with oscillatory saws. They found a higher degree of formation of vascularized tissues, of provisional matrix, and of bone remodeling activity at 7 and 14 days after use of piezoelectric surgery. It could be hypothesized that the bur damages the surrounding bone and interferes with the demineralization process of the early phase of the RAP.

From a macromorphological standpoint, the cortical layer of the defect side of PTP samples (Figure 4) appears thicker than the cortical layer of other samples. This effect has been discussed in other studies and is believed to be a result of ultrasonic vibrations leading to cell activation, inducing new bone deposition (Esteves et al., 2013; Fujiwara et al., 2021; Tunçer et al., 2018).

Male rats only were used in an attempt to standardize as much as possible the biological modifications in bone. Female rats have been known to have a slightly different osseous physiology than males (Hefferan et al., 2003).

Limitations of this pilot study includes small group sizes, the use of long bones instead of facial bones (endochondral vs. intramembranous ossification) and the fact that only the early phase of the healing was observed. Only one factor (Ct.Po) was measured, and no longitudinal data was obtained. The nature of this study (imaging‐based) also precludes us from analyzing the content of the porosities. Because the segmentation algorithms were trained on hand‐segmented slices, there is potential for a systematic “error of the method.” A study of a longer duration with a larger sample size as well as systemic comparison with histology would be necessary to assess the contents and evolution of these porosities.

These results must be interpreted cautiously, as this article reports the results of a pilot study on the animal model. The experiments were conducted on the tibia, which may react differently to these injuries than the maxilla or the mandible. Further investigations on the subject are warranted and should be conducted before these findings can be directly extrapolated to a clinical scenario.

The results obtained in this study suggests that TPs using a piezoelectric knife induces a statistically significant more pronounced RAP than using a rotary bur or with reflection of tissues alone in the early stages of wound healing in the rat model.

## AUTHOR CONTRIBUTIONS

Drs. Serge Dibart, Massimo Di Battista, and Taisuke Ohira designed the experiment. Drs. Elias Exarchos, Taisuke Ohira, and Massimo Di Battista conducted the surgical procedures. Drs. Elias Exarchos, Taisuke Ohira, and Massimo Di Battista performed the sample preparation and image acquisition. Dr. Massimo Di Battista made the imaging analysis. Drs. Serge Dibart, Taisuke Ohira, Massimo Di Battista, Jeremy Kernitsky, and Elias Exarchos performed the statistical analyses and data interpretation.

## CONFLICT OF INTEREST

The authors declare no conflict of interest.

## Data Availability

The data that support the findings of this study are available from the corresponding author upon reasonable request.
